# 

**Published:** 1857-01

**Authors:** 


NORTH AMERICAN

### MEDICO-CHIRURGICAL REVIEW.

NO. 1.—JANUARY, 1857.

### CONTENTS.

### ANALYTICAL AND CRITICAL REVIEWS.

PAGE

ART. I.—1. The Medical Profession of Ancient Times. By JOHN WATSON, M.D., Surgeon of the New York Hospital, . . . . . 1

2. History of Medicine from its origin to the nineteenth century. By F. V. RENOUARD, M.D. Translated by C. G. Comegys, M.D., 1

3. Recherches Historiques sur l'Exercise de la Médecine dans les Temples chez les Peuples de l'Antique, &c. Par L. P. AUGUSTE GAUTHIER, D.M.P., . . . . . . . . . . 1

ART. II.—A Treatise on the Practice of Surgery. By HENRY H. SMITH, M.D., Professor of Surgery in the University of Pennsylvania, . 26

ART. III.—Cretins and Cretinism. By GEO. S. BLACKIE, M.D., Curator of the Botanical Society of Edinburgh, . . . . . . 38

ART. IV.—The Transactions of the American Medical Association, Vol. IX. 1856, . . . . . . . . . . . . . 44

ART. V.—New Elements of Operative Surgery. By A. L. M. VELPEAU. Fourth edition with additions. By GEO. C. BLACKMAN, M.D., Professor of Surgery, Medical College of Ohio, . . . . 57

ART. VI.—Lectures on Materia Medica and Therapeutics. By JOHN B. BECK, M.D., Professor of Materia Medica and Therapeutics. Prepared for the press by C. R. GILMAN, M.D., Professor of Obstetrics in the College of Physicians and Surgeons at New York, . 60

ART. VII.—Transactions of the Indiana State Medical Society, at its Seventh Annual Session, . . . . . . . . . . . 61

ART. VIII.—A Treatise on Therapeutics and Pharmacology, or Materia Medica. By GEORGE B. WOOD, M.D., Professor of Medicine in the University of Pennsylvania, . . . . . . . . 65

### ORIGINAL COMMUNICATIONS.

ART. I.—Granular Conjunctiva. By C. S. FENNER, M.D., of Memphis, Tenn., 66

ART. II.—Management of Transverse Presentations of the Fœtus. By JOHN HARDIN, M.D., Professor of Obstetrics in the Kentucky School of Medicine at Louisville, . . . . . . . 73

ART. III.—On the Ligation of Arteries. By T. P. GIBBONS, M.D., . . . 78

ART. IV.—On the Economical Use of Quinia. By O. C. GIBBS, M.D., of Chatauque Co., New York, . . . . . . . . 79

ART. V.—A New Plan for the Treatment of Uterine Hemorrhages. By M. DUPIERRIS, of Havana, . . . . . . . . . 83
ART. VI.—Account of a New Tourniquet. By S. D. GROSS, M.D., Prof. of Surgery in the Jefferson Medical College of Philadelphia, . 96
ART. VII.—On the Evil Effects of Marriages of Consanguinity. By S. M. BEMISS, M.D., of Louisville, Ky., . . . . . . 97

BI-MONTHLY PERISCOPE.

PHYSIOLOGY.

1. Obliteration of the Portal Vein, . . . . . . . . . . 108
2. Temperature of the Blood lowered by passing through the Lungs, . 109
3. Coagulation of the Blood, . . . . . . . . . . 109

SURGERY.

1. Case of Laceration of the Perineum successfully operated upon, . . 110
2. Chronic Abscess of the Tibia, . . . . . . . . . . 113
3. Mode of reducing Dislocated Thumb, . . . . . . . . 113
4. Treatment of Ranula, . . . . . . . . . . . 114
5. Injection of Balsam Copaiba in Gonorrhœa, . . . . . . 114
6. Perchloride of Iron as a Hemostatic, . . . . . . . . 115
7. Surgical Statistics of Massachusetts General Hospital, . . . 115
8. Perchloride of Iron in Panniform Keratitis, . . . . . . 117
9. Successful Ligature of the Innominate Artery, . . . . . . 117
10. Glycerine and Tannin in Vaginitis, . . . . . . . . 118
11. Inverted Nail cured by the local application of Solution of Acetate of Lead, . . . . . . . . . . . . . . 119
12. Spina Bifida, . . . . . . . . . . . . . 120
13. Traumatic Tetanus cured by local application of Chloroform to the Spine, . . . . . . . . . . . . . 121

PRACTICAL MEDICINE, ETC.

1. Diagnosis of Phthisis by the Microscope, . . . . . . . 123
2. Treatment of Chorea by Gymnastics, . . . . . . . . 125
3. Large Intestinal Calculus, . . . . . . . . . . 127
4. Pericarditis, . . . . . . . . . . . . . 128
5. Idiopathic Tetanus; two Cases, with one Case after Abortion, and Notes on Traumatic Tetanus, . . . . . . . . . . 130
6. Charcoal in Epidemics of Measles and Cholera, . . . . . 132

OBSTETRICS, ETC.

1. Carbonic Acid as a local Anæsthetic in Uterine Diseases, . . . 137
2. Effect of Carbonic Acid on the Gravid Uterus, . . . . . . 139
3. Carbonic Acid as a Means of producing Premature Labor, . . . 139
4. Stricture of the Uterus, . . . . . . . . . . 140
5. Criminal Abortion—Peritonitis, . . . . . . . . . 142
6. Development and Decline of the Function of Menstruation, . . . 142
7. Abortion the Result of patulous Condition of the Os Uteri, . . . 145
8. Rules for the Resuscitation of Still-Born Infants, . . . . . 146
EDITORS' TABLE, . . . . . . . . . . . . . 148
BI-MONTHLY BIBLIOGRAPHICAL RECORD, . . . . . . . . 160

